# Comparing a disease-specific and a generic health-related quality of life instrument in subjects with asthma from the general population

**DOI:** 10.1186/1477-7525-6-15

**Published:** 2008-02-15

**Authors:** Milo A Puhan, Jean-Michel Gaspoz, Pierre-Olivier Bridevaux, Christian Schindler, Ursula Ackermann-Liebrich, Thierry Rochat, Margaret W Gerbase

**Affiliations:** 1 Department of Internal Medicine, Horten Centre for Patient-oriented Research, University Hospital of Zurich; CH-8091 Zurich, Switzerland; 2 Department of Health and Community Medicine, Division of Community and Primary Care Medicine, University Hospitals of Geneva, CH-1211 Geneva 14, Switzerland; 3 Department of Internal Medicine, Division of Pulmonary Medicine, University Hospitals of Geneva, CH-1211 Geneva 14, Switzerland; 4 Institute of Social and Preventive Medicine, University of Basel, CH-4051 Basel, Switzerland

## Abstract

**Background:**

Few epidemiologic studies have assessed health-related quality of life (HRQL) of asthma patients from a general population and it is unclear which instrument is best suitable for this purpose. We investigated the validity of the Asthma Quality of Life Questionnaire (AQLQ) and the SF-36 completed by individuals with asthma from the population-based SAPALDIA (Swiss study on air pollution and lung diseases in adults) cohort.

**Methods:**

The study included 258 participants with a physician-diagnosed asthma who had completed the AQLQ and SF-36. We assessed floor and ceiling effects, internal consistency reliability and cross-sectional validity with *a priori *hypotheses that correlations between the specific HRQL domains (e.g. "symptoms" or "physical functioning") and the corresponding external validation measures (respiratory symptoms, need for doctor visits, limitation in activities due to asthma and lung function) would capture similar aspects and be correlated moderately (≥ 0.3) to strongly (≥ 0.5), whereas non-corresponding domains be correlated weakly with each other (<0.3).

**Results:**

The AQLQ showed pronounced ceiling effects with all median domain scores above 6 (scores varied from 1–7). For the SF-36, ceiling effects were present in 5 out of 8 domains. Cronbach's alpha was >0.7 for all AQLQ and SF-36 domains. Correlations between the AQLQ domains "respiratory symptoms", "activity limitation" and "environmental exposure", and the validation measures ranged from 0.29–0.57. Correlations between the "emotional function" domain and the validation measures were also in this range (0.31–0.55) and not as low as we hypothesized. For the SF-36, correlations between "physical functioning" and "role physical", and the validation measures ranged from 0.25–0.56, whereas "role emotional" and "mental health" correlated with these measures from 0.01–0.23.

**Conclusion:**

The AQLQ and the SF-36 showed fairly good internal consistency. Both instruments are limited by ceiling effects, but they appear less pronounced in the SF-36, which also shows a better discrimination between different aspects of HRQL. The SF-36 may therefore be a more valid measure of HRQL than the AQLQ when applied to individuals with asthma from the general population.

## Introduction

In asthma research, there is growing evidence indicating that traditional outcomes, such as respiratory symptoms and pulmonary function, do not entirely express the patients' perception of the limitation determined by this condition [1]. Assessment of health-related quality of life (HRQL) has become central to assess the self-perceived impact of physical and mental impairment on patients' health [2]. However, reports on HRQL from subjects with asthma from the general population are scarce.

To date, more than a hundred randomised trials have reported the beneficial effects of a variety of therapeutic interventions to reverse airway obstruction and respiratory symptoms in patients with asthma, but very few studies have assessed HRQL parameters [3-5]. In addition, HRQL has been rarely investigated in non selected individuals with asthma from the general population, who may differ substantially from patients selected for clinical trials [6].

To assess HRQL of the universe of subjects with asthma, observational population-based studies are by design less prone to the restrictive selection criteria of clinical trials and are more suitable to provide valid effect estimates that can be applied to the "real" world setting [7-9]. A requirement is, however, to have a valid HRQL instrument. One of the most widely used instruments to assess self-perceived asthma is the disease-specific Asthma Quality of Life Questionnaire (AQLQ)[10]. Disease-specific instruments are known to be more sensitive to assess changes within patients [11,12]. On the counterpart, generic instruments such as the Short-Form Survey 36 (SF-36) are less sensitive to assess intra-individual changes, but are specifically designed to detect differences between individuals from a general population [13].

It is unclear whether the AQLQ or the SF-36 is the best choice for asthma patients enrolled in population-based studies. The measurement properties of the HRQL instruments depend substantially on the population under study. Therefore, the aim of our study was to investigate the measurement properties of the AQLQ and the SF-36 completed by individuals with asthma from the population-based SAPALDIA (Swiss Study on Air Pollution and Respiratory Diseases in Adults) cohort.

## Methods

### A priori considerations

We postulated that, in order to be useful for epidemiologic studies, the AQLQ or SF-36 should fulfil the following requirements. First, the AQLQ and SF-36 should discriminate well amongst patients with different degrees of impairment. For this specific purpose they should show a wide distribution of scores with low ceiling effects and good cross-sectional validity. Second, the domain scores of the questionnaire should adequately express the information contained in its single items. For this purpose, we analysed measures of internal consistency reliability. Finally, we assessed the acceptability of the questionnaires by calculating the extent of missing items within each HRQL instrument. The analysis of measurement properties requiring a longitudinal assessment were beyond the scope of this study.

### The SAPALDIA study

We reported on the methods of the SAPALDIA cohort study in detail previously [14,15]. Briefly, the SAPALDIA cohort constitutes of a random sample of 9651 adults recruited initially in 1991. Eight study areas (Geneva, Basel, Lugano, Aarau, Wald, Payerne, Davos, Montana) were chosen to represent the geographic, environmental and cultural diversity of Switzerland. A random sample of persons aged 18–60 years, who had been residents in the respective area for at least three years, were drawn from the local registries of inhabitants of these areas. Health examinations were conducted at the eight local centres at baseline in 1991 and at follow-up in 2002. Subjects answered an interview-based standardised questionnaire, adapted from the European Community Respiratory Health Study [16] and to the Short-form 36 questionnaire [13]. In addition, participants with asthma were invited to complete the Asthma Quality of Life Questionnaire [10]. Spirometry meeting the American Thoracic Society criteria was performed at both surveys. For this study, spirometry parameters (FVC, FEV_1 _and FEF_25–75_) obtained during the follow-up survey in 2002 were used for analyses. Ethical approval was obtained from the Swiss Academy of Medical Sciences and the Regional Ethics Committees. Written informed consent was obtained from all participants at both surveys.

### Inclusion criteria for analysis

We identified all adults with asthma from the SAPALDIA cohort who had completed the AQLQ at the second survey in 2002. The diagnosis of asthma was based on the answer *"yes" *to the following questions: "*Do you have asthma?" *and "*Has your asthma been diagnosed by a doctor?"*

### HRQL instruments

The AQLQ is a disease-specific instrument and addresses physical and mental impairment commonly related to asthma [10]. It consists of four domains "symptoms" (12 items), "activity limitation" (11 items), "emotional function" (5 items) and susceptibility to "environmental exposure" (4 items). Domain scores are calculated as the average of the items within each domain and presented on a Likert-type scale varying from 1 (most severe impairment) to 7 (no impairment).

The SF-36 is the most widely used generic HRQL instrument and has been used frequently in observational studies as well as in clinical trials [13]. The 36 items cover a broad range of symptoms and limitations. The eight domain scores are generated from 2–10 items and expressed on a scale varying from 0–100. For some domains such as the "role physical" domain, scores range from 0–100 but only the scores 0, 25, 50, 75 and 100 are possible. Two summary scores (physical and mental component summary score) can be derived from the domain scores. The component summary scores are standardized (t scores) to have a mean value of 50 for the general population with a standard deviation of 10.

### Validation measures

We used a number of validation measures available from the SAPALDIA database that met the Global Initiative for Asthma criteria for the assessment of asthma [17]. Data from the SAPALDIA questionnaire included information on regular visits to medical doctors (general practitioner and pulmonologist) due to asthma-related symptoms during the 12 months preceding the SAPALDIA examination [18,19]. For "respiratory symptoms", answers on the presence of wheeze, cough (day or night), phlegm (day or night) and dyspnea were computed as a simple cumulative score varying between 0 and 4 to indicate the number of reported symptoms [20]. The SAPALDIA questionnaire includes a question about the avoidance of physical activity due to asthma ("exercise limitation"). Information about "professional and leisure limitation due to asthma" was covered by items such as inability to work, sick leaves or limitations of leisure time activities because of asthma. Other validation measures included information on the subjects' smoking status, spirometry results (percent of predicted FEV_1 _and FEF_25–75_), as well as patients' self-reported and physician-diagnosed depression. In the SAPALDIA survey, answers to symptom questions included 4 options (*yes*, *no*, *don't know *and *refuse to answer*). Missing answers were considered when none of the 4 options was selected by the participant.

### Statistical analysis

We first determined the extent of missing data for each item of the two questionnaires. We then assessed the distributions of the AQLQ and SF-36 domain scores graphically and by looking at skewness and kurtosis. Since AQLQ and SF-36 scores were skewed towards high scores and not distributed normally, we calculated medians and interquartile ranges (25^th ^to 75^th ^percentile) and used non-parametric tests.

In order to determine floor and ceiling effects, we calculated the percentage of patients with very low and very high scores. Since there is no consensus on how to define floor and ceiling effects mathematically, we determined *a priori *that floor and ceiling effects were present when the AQLQ domain scores were found between 1 and 2, and 6 and 7, respectively, and when the SF-36 domain scores were found between 0 and 10, and 90 and 100, respectively.

For internal consistency reliability, we used three measures: corrected item-total correlations, inter-item correlations and Cronbach's alpha. Corrected item-total correlations indicate the extent to which each item relates to the construct measured by the total score. Correcting the total score by removing the item of interest prevents spuriously high values due to item overlap. A recommended minimum value is 0.40 [21]. Inter-item correlations represent the mutual relation between individual items and should exceed 0.3 [21]. We also calculated the Cronbach's alpha coefficient to further assess internal consistency. Cronbach's alpha should exceed 0.7 [21]. In the absence of a gold standard, the most widely established method to investigate validity is the correlation approach, which allows to assess dissimilarities and complementarities between instruments [2,22]. To assess this, we hypothesized a priori that correlations (Spearman rank correlation coefficients) of specific AQLQ and SF-36 domains representing physical impairment (e.g. "symptoms" or "physical functioning") should correlate moderately (correlation coefficients ≥ 0.3) to strongly (≥ 0.5) with validation measures capturing similar aspects ("respiratory symptoms" or "professional and leisure limitation due to asthma"). Non-corresponding domains (e.g. AQLQ domain for "symptoms" and presence of "depression") should correlate weakly with each other (<0.3). Accordingly, AQLQ and SF-36 domains representing mood or psychological status ("emotional function" or "role emotional") should correlate moderately to strongly with self-reported or doctor-diagnosed "depression" and only weakly with validation measures for physical impairment ("respiratory symptoms" or "airflow obstruction"). We assumed cross-sectional validity to be sufficient if the observed correlations corresponded to our hypothesized correlations. Finally, we performed sensitivity analysis restricted to the subset of subjects who reported an exacerbation of asthma in the period preceding the SAPALDIA follow-up visit, to investigate whether the HRQL scores were lower and the cross-sectional validity was different in these individuals, as compared to the whole cohort. For the purpose of this analysis, subjects included in the subgroup with exacerbation of asthma were defined by the reports of regular use of asthma medication in the three months preceding the follow-up SAPALDIA survey in 2002 (SAPALDIA 2). We performed all statistical analyses using SPSS 12.01 for Windows (SPSS Inc, Chicago, Ill).

## Results

We identified a total of 615 individuals with asthma in the SAPALDIA cohort who participated in the second survey. Out of 615 subjects, we included 258 individuals (42%) in the analyses, who had completed the AQLQ. 216 of these subjects (84%) had also completed the SF-36. Table 1 shows the characteristics of subjects who had completed only the AQLQ, subjects who had completed both the AQLQ and the SF36, and subjects who had not completed any of the HRQL instruments. Percent of predicted FEV_1 _and FEF_25–75_of the participants included in the analyses were 85.3 ± 19.0% and 69.8 ± 32.1% (mean ± SD), respectively. Wheezing was reported by 60.7% of the subjects, whereas 24.0% or less reported dyspnea, cough or phlegm. The majority of subjects reported relatively few asthma attacks (median number of asthma attacks 3, interquartile range 1–12) during the 12 months preceding the SAPALDIA 2 assessment and 55.4% of subjects denied the use of asthma medication in the 3 months prior to the survey.

**Table 1:** Characteristics of participants of the SAPALDIA cohort according to the completion of the HRQL instruments

	**AQLQ** Group A*	**AQLQ + SF36** Group B*	**No AQLQ, no SF36** Group C*
Age, years ± SD	48.7 ± 14.0	47.2 ± 14.2	49.3 ± 15.5
Male gender, %	49.2	47.5	45.7
BMI, ± SD	22.3 ± 6.0	22.4 ± 6.3	20.9 ± 8.5
Low level of education, %	19.1	19.0	14.2*
Current smokers, %	17.2	17.6	27.2^†^
Formers smokers, %	31.7	33.5	33.3
Never smokers, %	50.8	48.9	37.0^‡^
Cough, %	20.2	16.7	27.2^§^
Phlegm, %	15.3	12.2	19.8
Wheeze, %	60.7	57.0	51.9
Dyspnea, %	24.0	21.7	18.5
Depression,%	12.2	12.7	13.0
Exercise limitation, %	24.8	24.3	8.6^€^
GP visits, %	34.7	33.9	27.8
Lung specialist visits, %	16.4	14.5	8.0^¥^
FVC, % of predicted value ± SD	94.9 ± 14.6	96.5 ± 13.3	94.7 ± 15.0
FEV_1_, % of predicted value ± SD	85.3 ± 19.7	87.1 ± 18.4	90.2 ± 16.5
FEF_25–75_, % of predicted value ± SD	69.8 ± 32.1	71.1 ± 30.8	81.7 ± 26.8
Use of asthma medication during last 3 months,%	38.8	32.5	12.3^€^
Number of asthma attacks in last 12 months, median (25^th ^to 75^th ^percentile)	3 (1–12)	3 (1–12)	3 (1–10)
Number of asthma attacks in last 3 months, median (25^th ^to 75^th ^percentile)	2 (1–7)	2 (1–6)	2 (1–4)

Group A: Participants who had completed the Asthma quality of life questionnaire (AQLQ) only; group B: participants who had completed the AQLQ and the short-form 36 questionnaire (SF36); group C: participants who had not completed the AQLQ nor the SF36.

BMI: body mass index; GP: general practitioner; FVC: forced vital capacity; FEV_1_: forced expiratory volume at one second; FEF_25–75_: forced expiratory flow measured between 25% and 75% of the forced vital capacity. *p = 0.01 and p < 0.05 compared to groups A and B, respectively; ^†^p = 0.01 and p < 0.05 compared to groups A and B, respectively; ^‡^p < 0.01 and p < 0.05 compared to groups A and B, respectively;^§^p = 0.01 compared to group B; ^€^p < 0.0001 compared to groups A and B;^¥^p = 0.01 and p = 0.05 compared to groups A and B, respectively.

To assess the extent of potential selection bias, Table 1 also shows the characteristics of participants who had either completed the AQLQ only or none of the HRQL instruments. We found that participants who refused to complete the HRQL instruments were more likely to be smokers and to present symptoms of cough, and less likely to be limited by exercise and to visit a lung specialist. Also, subjects who refused to complete the HRQL instruments had relatively little use of asthma medication (12.3% during the 3 months preceding the survey), whereas the number of asthma attacks was comparable to those completing HRQL instruments. In addition, we compared the presence of other co-morbidities between groups. Frequency of systemic hypertension (22% and 26%), cardio-vascular diseases (8% and 12%), diabetes (5% and 9%) and cancer (5% and 3%) in subjects who answered the quality of life questionnaires and subjects who refused to participate in these surveys, respectively, was comparable as well.

The occurrence of missing answers was low for both questionnaires. For the AQLQ the percentage of missing answers per item was below 3% for 31 of the 32 items. However, in one single item (number 5, "being troubled by asthma during the night") the rate of missing answers was 7%. For the SF-36, less than 3% missing answers was found for each of the 36 items.

**Figure 1:**
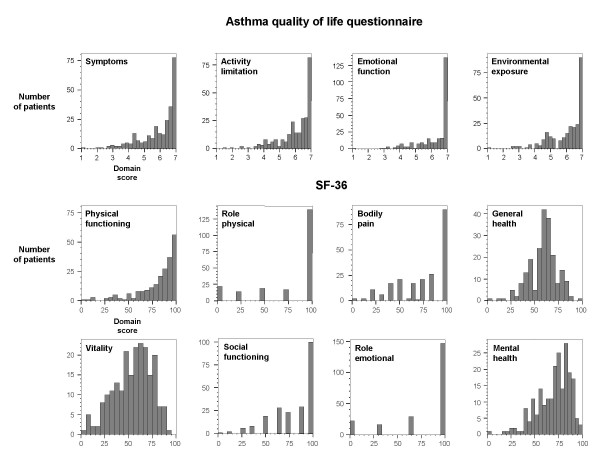
**Distribution of AQLQ and SF-36 domain scores**. The figure shows the distributions of the respondents' AQLQ (Likert type scale from 1 to 7) and SF-36 scores (from 0 to 100). For the AQLQ all domain scores are left-skewed. For the SF-36, the "physical functioning", "role physical", "bodily pain", "social functioning" and "role emotional" domains are left-skewed. The "general health", "vitality" and "mental health" domains showed almost normal distributions.

For the AQLQ domains, median scores were all above 6 and more than 75% of all respondents had scores above 5 (Table 2). Figure 1 shows this pronounced ceiling effect. For the SF-36, ceiling effects were also present in the "physical functioning", "role physical", "bodily pain", "social functioning" and "role emotional" domains. The "general health", "vitality" and "mental health" domains showed almost normal distributions.

**Table 2:** Scores of the AQLQ and the SF-36 in individuals with asthma from a general population

**Instrument**	**Number of items**	**Median scores (25th to 75th percentile)**	**Floor and ceiling effects**		**Internal consistency reliability**		
			% scores 1–2	% scores 6–7	Cronbach alpha	Corrected item total correlations	Inter-item correlation

**AQLQ **(n = 258)							
Symptoms	11	6.38 (5.33–6.92)	0.4	72.1	0.92	0.47–0.86	0.22–0.89
Activity limitation	12	6.36 (5.64–6.91)	0.4	60.9	0.95	0.64–0.86	0.40–0.79
Emotional function	5	6.80 (5.80–7.00)	0.4	64.0	0.84	0.53–0.76	0.37–0.70
Environmental exposure	4	6.50 (5.25–7.00)	0.4	66.7	0.77	0.50–0.64	0.34–0.56

			% scores 0–10	% scores 90–100			

**SF-36 **(n = 216)							
Physical functioning	10	90.0 (75.0–100.0)	2.3	54.8	0.92	0.57–0.81	0.30–0.82
Role physical	4	100.0 (50.0–100.0)	10.4	66.0	0.86	0.69–0.73	0.57–0.64
Bodily pain	2	84.0 (51.0–100.0)	1.4	41.7	0.93	0.88	0.88
General health	5	58.8 (50.0–69.1)	0.5	0.5	0.77	0.38–0.71	0.22–0.66
Vitality	4	60.0 (45.0–70.0)	2.8	3.7	0.88	0.71–0.79	0.51–0.78
Social functioning	2	87.5 (62.5–100.0)	0.5	46.3	0.80	0.74	0.74
Role emotional	3	100.0 (66.7–100.0)	10.3	68.7	0.78	0.55–0.67	0.48–0.65
Mental health	5	76.0 (60.0–84.0)	0.5	11.6	0.88	0.66–0.78	0.46–0.75
Physical component score		52.4.(44.1–56.4)					
Mental component score		50.8 (41.8–54.2)					

AQLQ: Asthma quality of life questionnaire; SF-36: Short-form 36 questionnaire

Cronbach alpha was above 0.7 for all AQLQ and SF-36 domains (Table 2). Corrected item-total and inter-item correlations were also high with few exceptions. Table 3 shows the correlations between the AQLQ and SF-36 that were, in general, moderate to high. Correlations were not lower between domains that are supposed to measure different constructs. For example, correlations of the "physical functioning" domain of the SF-36 were high with all four AQLQ domains.

**Table 3:** Correlation between the AQLQ and the SF-36

**AQLQ**	**Symptoms**	**Activity limitation**	**Emotional function**	**Environmental exposure**
**SF-36**				
Physical functioning	0.49	0.61	0.54	0.51
Role physical	0.32	0.42	0.34	0.33
Bodily pain	0.33	0.36	0.29	0.33
General health	0.32	0.41	0.30	0.36
Vitality	0.34	0.34	0.30	0.28
Social functioning	0.34	0.36	0.34	0.29
Role emotional	0.27	0.32	0.31	0.26
Mental health	0.36	0.33	0.30	0.31
PCS	0.40	0.51	0.41	0.42
MCS	0.26	0.19	0.21	0.18

Table 4 shows the correlations to investigate cross-sectional validity of the two questionnaires. Correlations of the "symptoms", "activity limitation" and "environmental exposure" domains of the AQLQ with "exacerbation of asthma", "respiratory symptoms", "exercise limitation" and "professional or leisure limitation" were moderate to high, as hypothesized. Correlations were similarly high for the "emotional function" domain and substantially higher than we hypothesized a priori. In turn, the AQLQ "emotional function" domain correlated only weakly with "depression" as did the "symptoms", "activity limitation" and "environmental exposure" domains.

**Table 4:** Cross-sectional validity for the entire group of subjects

**Instrument**	**AQLQ**							
**Validation measure**	**Activity limitation**	**Symptoms**	**Emotional function**	**Environmental exposure**				

**Severity of asthma**	-0.33	-0.41	-0.38	-0.34				
**Respiratory symptoms**	-0.57	-0.67	-0.55	-0.57				
**Exercise limitation**	-0.40	-0.32	-0.35	-0.29				
**Professional or leisure limitation due to asthma?**	-0.37	-0.35	-0.31	-0.34				
**Depression**	-0.17	-0.10	-0.09	-0.12				
**Smoking**								
**Current**	0.02	-0.08	-0.03	-0.06				
**Current or former**	-0.15	-0.10	-0.12	-0.10				
**FEV_1 _(% of predicted value)**	0.37	0.42	0.41	0.37				
**FEF_25–75 _(% of predicted value)**	0.31	0.21	0.26	0.24				

	**SF-36**							

	**Physical functioning**	**Role physical**	**Bodily pain**	**General health**	**Vitality**	**Social functioning**	**Role emotional**	**Mental health**

**Severity of asthma**	-0.31	-0.25	-0.20	-0.31	-0.14	-0.19	-0.14	-0.14
**Respiratory symptoms**	-0.37	-0.27	-0.28	-0.32	-0.30	-0.26	-0.21	-0.23
**Exercise limitation**	-0.39	-0.26	-0.19	-0.18	-0.24	-0.15	-0.13	-0.13
**Professional or leisure limitation due to asthma?**	-0.30	-0.32	-0.25	-0.35	-0.22	-0.25	-0.21	-0.13
**Depression**	-0.16	-0.24	-0.25	-0.20	-0.29	-0.25	-0.31	-0.27
**Smoking**								
**Current**	0.03	0.05	-0.04	0.03	0.01	0.00	0.08	-0.02
**Current or former**	-0.07	-0.10	-0.13	0.09	-0.05	-0.08	0.01	0.01
**FEV_1 _(% of predicted value)**	0.56	0.32	0.23	0.20	0.16	0.20	0.21	0.10
**FEF_25–75 _(% of predicted value)**	0.45	0.30	0.28	0.12	0.07	0.14	0.13	0.02

The SF-36 domains assessing physical impairment ("physical functioning" and "role physical") correlated substantially higher with the external validation measures than the domains evaluating mental impairment ("role emotional" and "mental health"). On the other hand, "vitality", "role emotional" and "mental health" correlated better with "depression" than the SF-36 domains assessing physical impairment.

In the subgroup of subjects defined as having an exacerbation of asthma at the interview in 2002 (n = 143), the AQLQ domain scores were lower (median 5.75 [interquartile range 4.58–6.67] for "symptoms", 6.00 [5.09–6.72] for "activity limitation", 6.20 [5.20–6.80] for "emotional function" and 6.00 [4.75–6.75] for "environmental exposure") than in the overall cohort (median scores of 6.38, 6.36, 6.80 and 6.50, respectively, see Table 2). The SF-36 scores were also lower for this sub-sample of individuals for some domains (85.0 [63.8–95.0] for "physical functioning", 74.0 [51.0–100.0] for "bodily pain", 55.0 [40.0–70.0] for "vitality" and 75.0 [62.5–100.0] for "social functioning") while they were similar for the other domains (100.0 [50.0–100.0] for "role physical", 58.8 [46.3–65.0] for "general health", 100.0 [66.7–100.0] for "role emotional" and 76.0 [56.0–84.0] for "mental health").

Table 5 shows, however, that the correlation coefficients comparing domains from the two instruments and the external validation measures were not substantially different from the correlation coefficients calculated for the whole study population. For example, correlation coefficients between the AQLQ "activity limitation" domain and the several validation measures differed by less than 0.1; similarly, the correlation coefficients for the whole cohort (Table 4) differed by ≤ 0.1 when compared to the coefficients obtained for subjects classified as having and exacerbation of asthma (Table 5).

**Table 5:** Cross-sectional validity for individuals with an exacerbation of asthma at the follow-up survey* (n = 143)

**Instrument**	**AQLQ**							
**Validation measure**	**Activity limitation**	**Symptoms**	**Emotional function**	**Environmental exposure**				

**Severity of asthma**	-0.27	-0.34	-0.33	-0.28				
**Respiratory symptoms**	-0.49	-0.62	-0.43	-0.52				
**Exercise limitation**	-0.32	-0.39	-0.32	-0.30				
**Professional or leisure limitation due to asthma?**	-0.34	-0.34	-0.24	-0.30				
**Depression**	-0.21	-0.13	-0.09	-0.15				
**Smoking**								
**Current**	-0.05	-0.10	-0.08	-0.13				
**Current or former**	-0.08	-0.21	-0.14	-0.14				
**FEV_1 _(% of predicted value)**	0.34	0.27	0.34	0.29				
**FEF_25–75 _(% of predicted value)**	0.24	0.12	0.21	0.18				

	**SF-36**							

	**Physical functioning**	**Role physical**	**Bodily pain**	**General health**	**Vitality**	**Social functioning**	**Role emotional**	**Mental health**

**Severity of asthma**	-0.24	-0.25	-0.18	-0.25	-0.03	-0.16	-0.12	-0.12
**Respiratory symptoms**	-0.38	-0.29	-0.31	-0.26	-0.30	-0.20	-0.20	-0.29
**Exercise limitation**	-0.42	-0.20	-0.19	-0.16	-0.27	-0.10	-0.11	-0.18
**Professional or leisure limitation due to asthma?**	-0.34	-0.38	-0.28	-0.32	-0.19	-0.20	-0.22	-0.15
**Depression**	-0.26	-0.25	-0.33	-0.38	-0.27	-0.38	-0.35	-0.25
**Smoking**								
**Current**	0.00	0.02	-0.11	0.01	0.03	0.04	0.09	0.04
**Current or former**	-0.11	-0.12	-0.23	0.11	-0.14	-0.12	-0.01	0.03
**FEV_1 _(% of predicted value)**	0.57	0.32	0.18	0.12	0.06	0.18	0.21	0.05
**FEF_25–75 _(% of predicted value)**	0.48	0.33	0.30	0.09	0.06	0.21	0.23	0.03

* Selection criteria are described in the Methods section

## Discussion

Our findings show that cross-sectional validity, expressed by the correlations between the SF-36 and AQLQ domains and the validation measures, approached the *a priori *hypothesized correlations for the SF-36 and, to a lesser extent, for the AQLQ. Both instruments showed good internal consistency reliability. There were few missing items on the completed questionnaires, which indicates satisfactory acceptability. However, both instruments showed important ceiling effects, which seemed less marked for the SF-36.

To our knowledge, this is the first study to assess the measurement properties of the AQLQ and SF-36 in asthma patients of a general population. The AQLQ discriminated between patients with different disease severity as shown by the lower domain scores found in patients presenting an exacerbation of the disease and requiring regular use of asthma medication. But the different domains did not discriminate well between physical and emotional functioning domains covered by this instrument. As such, correlations of the four AQLQ domains with validation measures were similar although they are conceived to capture different aspects of HRQL.

Emotional function may be impaired in asthma patients as a consequence of physical impairment and, therefore, may also correlate with measures capturing physical impairment. However, one would still expect lower correlations than between the "symptoms" and "activity limitation" domains and validation measures for physical impairment. If the different domains do not distinguish between different aspects of HRQL one might argue that the AQLQ is a valid measurement of overall HRQL but that the domains provide redundant information. In contrast to the AQLQ, we observed lower correlations between the SF-36 "role emotional" and "mental health" domains and the validation measures for physical impairment. Thus the SF-36 discriminates better between different aspects of HRQL. A likely explanation for the lower validity of the AQLQ is the pronounced ceiling effect. Subjects of this study were, on average, barely affected by asthma symptoms and inter-individual variability was low with limited scope for discrimination between patients.

Earlier validation studies of the AQLQ included asthma patients from clinical settings, where AQLQ scores were substantially lower and where no ceiling effect was present [10,23-26]. However, correlations of the AQLQ "emotional functioning" and the "symptoms" or "activity limitation" domains with validation measures were again similar. Our results are in line with these studies also showing that the AQLQ did not capture different aspects of HRQL in clinical populations.

Given the current evidence on the cross-sectional validity of the AQLQ, it appears that this instrument discriminates well between different degrees of asthma severity, but the specific domains addressed by the instrument do not discriminate well between different aspects of HRQL. The reason for this remains unclear. The development and the validation process of the AQLQ was identical to that of the Chronic Respiratory Questionnaire, a widely used disease-specific HRQL instrument for patients with chronic obstructive pulmonary disease [2]. The Chronic Respiratory Questionnaire also discriminates well between patients with different disease severity but its domains also capture different aspects of HRQL [27-29]. For example, the "dyspnea" domain correlates substantially better with the SF-36 "physical component summary score" than the "mental component summary score", and the "emotional function" domain correlates better with the SF-36 "mental component summary score" than the "physical component summary score".

In general, disease-specific instruments have been reported substantially more responsive than generic instruments and may be therefore more suitable to assess the disease impact on HRQL [11,12]. The SAPALDIA cohort does not provide data to assess responsiveness of the AQLQ and the SF-36. However, we could speculate that in a general population such as our cohort, the AQLQ would have similar responsiveness to the SF-36 due to the ceiling effects. Thus, in population-based studies it is, in general, difficult to assess changes over time. One possibility to overcome some of the ceiling effect in population-based studies might be to modify or increase the number of answer options to each question addressed by the HRQL instruments. Thereby, persons with mild disease would be able to express minimal degree of impairment or recovery. However, such modifications would require careful validation before being suitable for application in epidemiologic studies.

A limitation of our study is that only 258 participants completed the AQLQ, who represent approximately 40% of all subjects with asthma from the SAPALDIA cohort participating in the second survey. While comparing the characteristics of participants who completed and did not complete the HRQL instruments, we found that participants who did not complete the HRQL questionnaires were essentially less affected by asthma. It is, therefore, likely that inclusion of these participants would have led to even more pronounced ceiling effects. Another limitation is that we had only one external validation measure for psychological impairment. We did not have instruments such as the Hospital Anxiety and Depression Scale or similar instruments. However, self-reported depression was validated by an affirmative answer to a question on doctor-diagnosed depression.

Potential strengths of our study are the number of external validation measures for physical impairment and disease severity, and the approach to investigate validity. The highest level of evidence about an instrument's validity is generated when the observed correlation coefficients meet *a priori *hypothesized correlation results rather than just looking at the correlations [2]. Moderate to strong correlations *per se *may not represent sufficient validity. In our study, unexpectedly high correlations existed between the "emotional functioning" domain and the "severity of asthma" or "respiratory symptoms" domains. If correlations between such measures are found to be moderate to strong, although they are supposed to assess different aspects of health, it is unclear what they actually measure.

## Conclusion

The results of this study show that the AQLQ and SF-36 have good internal consistency. Both instruments are limited by ceiling effects, but the negative consequences on cross-sectional validity appear to be less pronounced for the SF-36. Therefore, the SF-36 seems a more suitable choice to assess HRQL in individuals with asthma from the general population.

## Competing interests

The author(s) declare that they have no competing interests.

## Authors' contributions

MAP participated in the design of the study, performed the statistical analysis and drafted the manuscript. JMG participated in the design of the study and revised the manuscript. POB participated in the design of the study and revised the manuscript. CS participated in the design of the study, reviewed the statistical analysis and revised the manuscript. UAL participated in the design of the study and revised the manuscript. TR participated in the design of the study and revised the manuscript. MWG participated in the design of the study, performed the statistical analysis and revised the manuscript. All authors read and approved the final manuscript.
